# Cutaneous manifestations of NAXD deficiency – A case report

**DOI:** 10.1016/j.amsu.2020.11.026

**Published:** 2020-11-07

**Authors:** Mohammad Umair Malik, Haleema Nadir, Zita Maria Jessop, Jonathan James Cubitt

**Affiliations:** a The Welsh Centre for Burns and Plastic Surgery, Morriston Hospital, Heol Maes Eglwys, Morriston, Cwmrhydyceirw, Swansea, SA6 6NL, United Kingdom; b Reconstructive Surgery and Regenerative Medicine Research Group Swansea University Medical School, Swansea, SA2 28PP, United Kingdom; c Barts and the London School of Medicine and Dentistry, Whitechapel, London, E1 2AD, United Kingdom

**Keywords:** NAXD deficiency, Neurodegenerative disease, Metabolic disorder, Paediatric disease, Novel, Case report

## Abstract

Metabolism is a tightly regulated sequence of events, supported by key reactions between enzymes and enzyme-specific substrates. These reactions have the potential to produce metabolic side products that can have deleterious effects to further key metabolic reactions. The nicotinamide repair system consists of two partner enzymes, NAD(P)HX epimerase (NAXE) and NAD(P)HX dehydratase (NAXD). These enzymes regulate the levels of metabolic side products. Here we present a case of an 11-month old child who presented to our paediatric department with pyrexia, lethargy and multiple cutaneous lesions on the background of NAXD deficiency, a lethal neurometabolic disorder of early childhood. Despite early intervention with intravenous antibiotics, the patient failed to improve and subsequently passed away. The skin lesions were thought to be a consequence of systemic disease rather than a propagator of infection. Clinicians should be aware of this incredibly rare metabolic disease, its potential to cause widespread systemic dysfunction and the developing avenues for management.

## Highlights

• Physical stress, infection or pyrexia have the potential to produce NADPHX.
• NADPHX is a metabolite with the ability to inhibit biosynthesis pathways.
• NAXE and NAXD are two cofactor enzymes that break down this toxic metabolite.
• NAXD deficiency is a rare metabolic disorder with potentially fatal outcomes
• Here we present a rare case of NAXD deficiency and its cutaneous manifestations.

## 1 Introduction

This case report adheres to the 2018 SCARE guidelines [1].

Metabolism is a tightly regulated sequence of events, supported by key reactions between enzymes and enzyme-specific substrates [2]. These reactions have the potential to produce metabolic side products, the accumulation of which can have potential deleterious effects to further key metabolic reactions. In 2013, Linster et al. [3] identified a number of enzymes forming integral metabolite repair systems in all domains of life.

Nicotinamide adenine dinucleotides (NAD) (reduced from NADH oxidised from NAD+) and nicotinamide adenine dinucleotide phosphate (NADP) (reduced from NADPH and oxidised from NADP+) have essential roles in a series of catabolic reactions, mitochondrial energy production and antioxidant protection systems [4,5]. NAD and NADP are prone to hydration, forming inactive cofactors NADHX and NADPHX, respectively [6]. This can occur spontaneously but may also be enzyme mediated.

Physical stress, infection or pyrexia also have the potential to produce NADPHX, a metabolite with the ability to inhibit biosynthesis pathways [6]. Its accumulation is toxic to cells, requiring a metabolite repair system to aid detoxification. The nicotinamide repair system is coordinated by two cofactor enzymes, NAD(P)HX epimerase (NAXE) and NAD(P)HX dehydratase (NAXD). NAXE converts R-NADPHX to S-NADPHX and NAXD which converts S-NADPHX back to NADPH in an ATP-dependant manner. NAXE and NAXD have a ubiquitous tissue distribution and are expressed across multiple species, suggesting they are a critical in sustaining life [7,8]. The literature reports a handful of cases in which a deficiency of this cofactor system has led to the development of significant multi-system dysfunction, such as rapid neurological deterioration, seizures, respiratory insufficiency and heart failure. Although the devastating effects on the brain and heart are well described, cutaneous manifestations are less widely reported [2,9,10]. Here we present a case of a child with NAXD deficiency, referred to our department with multiple cutaneous lesions on his back, axilla and nappy area. This case report aims to highlight the cutaneous manifestations of NAXD deficiency and stress the importance of early management of infection in patients with a background of this metabolic disorder.

## 2 Presentation of case

**Fig. 1:**
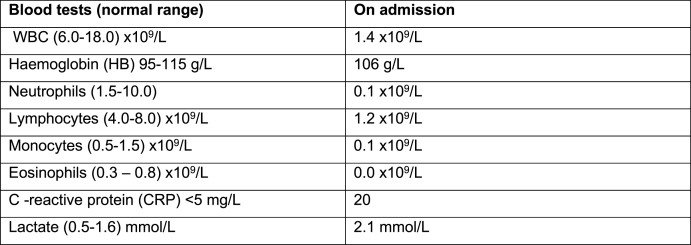
Blood tests on admission.

Baby X is a 11 month-old baby who presented to our paediatric department via ambulance with fevers, lethargy and loose stools. He had a past medical history of developmental delay, infantile spasms and seizures on the background NAXD deficiency. There was no family history of any metabolic disorders. The patient was pyrexial and leukopenic on admission (Fig. 1). He had been off his feeds and had a change in bowel habit for almost 2 weeks with an increasing frequency of spasms and seizures.

**Table:**

**Fig. 2:**
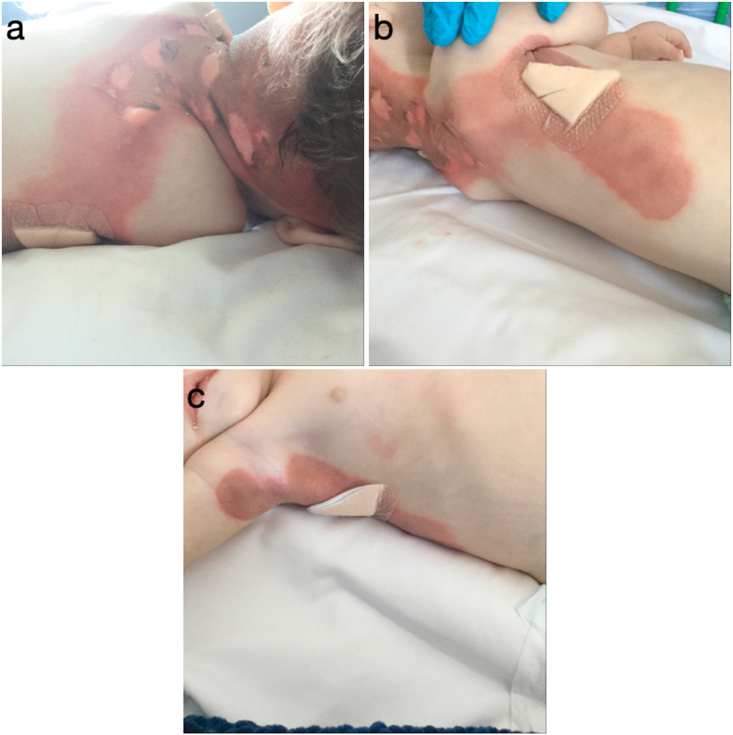
Figure containing images of Baby X, posterior trunk (A + B) and right axilla (C).

On admission, multiple skin lesions were noted, starting on the nape of the neck and progressing to the posterior aspect of the trunk, both axilla and nappy area (Fig. 2). These were confluent areas of epidermal loss and blistering. The clinical appearance was very similar to that of a superficial partial thickness burn covering approximately 5% TBSA and hence the patient was referred for Burns Centre Specialist review.

These were originally thought to be the potential cause of the patient's deterioration. The patient was treated for sepsis secondary to cellulitis, receiving intravenous broad-spectrum antibiotics including ceftriaxone 560mg once-daily and co-amoxiclav 210mg eight hourly (dose-adjusted for weight). It was later thought that these skin lesions were a consequence of systemic disease rather than the propagator of infection. These lesions were managed conservatively with a non-adhesive silicone-based dressing (Silflex©). Other options included simple paraffin, to which the patient was allergic.

The patient was thoroughly investigated for other potential sources of infection, including blood cultures, urinalysis, wound swabs, CT and MRI imaging of the brain. Urinalysis revealed a *Klebsiella* urinary tract infection. The patients’ antibiotic therapy was subsequently rationalised to cefalexin 175mg four times a day. Despite ongoing treatment, the combination of infection on the background of NAXD deficiency resulted in irreversible neurological damage. The patient failed to improve and subsequently passed away.

## 3 Discussion

NAXD deficiency is an incredibly rare mitochondrial disease that presents in early childhood, with only 18 cases recognised in the literature to date [2,10]. In this article, we present a rare case of systemic deterioration in a child with NAXD deficiency secondary to a Klebsiella urinary tract infection.

NAXD and NAXE are critical in the repair of toxic metabolites generated from NAD and NAPD, which have the potential to impede normal metabolic reactions critical to normal cellular function and overall health in humans. These disturbances occur through the inhibition of key cellular enzymes, including various mitochondrial dehydrogenases [2].

In 2019, Van Berger et al. [2] presented a series of individuals affected by homozygous and compound heterozygous NAXD mutations. As in this case, they observed multi-system disease and skin lesions induced by febrile illness. They report that the distribution of these lesions were similarly associated with areas of relatively high surface temperature, such as flexural creases (axillar and groin) and posterior neck [2,10]. Other notable clinical features associated with these episodes, include developmental regression and early onset neurodegeneration [2]. Earlier studies performed by Kremer et al. [9], using exome sequencing, identified biallelic pathogenic mutations in NAXE in children from four families suffering from sub-acute early-onset ataxia, cerebellar oedema, spinal myopathy and skin lesions. Like NAXD deficiency, these symptoms were precipitated and exasperated by fevers, ultimately leading to death in the early years of life [2,9].

NAXE levels in fibroblasts isolated from all four individuals were undetectable, with high concentrations of cyclic-NADHX produced from the degradation of NAD(P)HX, confirming deficiency of the NAD(P)HX cofactor system [9]. Fibroblasts harvested from patients with missense NAXD mutations also express greater levels of toxic co-factors, S-NADHX, R-NADHX and their derivative cyclic-NADHX, compared to the control. Furthermore, growth rates of fibroblasts harvested from individuals with NAXD deficiency showed significant decreased growth rates, when compared to control samples [1]. Both NAXD and NAXE deficient fibroblast populations demonstrate significantly reduced thermostability compared to the controls [2,7,9]. This, alongside increased spontaneous hydration of co-factors NADH and NADPH at increased temperatures and under acidic conditions provides some explanation as to why fever or infection can have such a detrimental effect [11,12]. These effects are more pronounced in the brain, where there is a sustained high energy demand and so, accumulation of toxic NAD(P)HX has particularly deleterious effects through disturbances in mitochondrial energy production [2]. The cutaneous effects of toxic cofactors appear to lead the disruption of the dermal-epidermal junction and this was confirmed by skin biopsies in previous reports, which showed detachment of epidermis from dermis with lymphocytes around vessels in the upper dermis [10]. We postulate that the cause of this may be due to local accumulation of toxic cofactors due to high surface temperature. Patients who present with pyrexia on the background of NAXD deficiency can be challenging to manage. It is important to recognise that infection can propagate multisystem dysfunction in patients with this metabolic disorder, requiring clinical input from various specialties. A multidisciplinary approach is necessary to provide appropriate support and management of vital organ systems.

Vitamin B3 (nicotinamide) therapy may provide a treatment option for relapse episodes in children suffering from nicotamide repair enzyme deficiencies. Although only reported in a single case, high dose vitamin B3 (500 mg/day) in a child with NAXD deficiency improved skin lesions and prevented further neurological decline [9].

## 4 Conclusion

NAXD deficiency is a multi-system disorder due to the enzymes ubiquitous expression. Patients suffering from this condition demonstrate a range of symptoms in the presence of pyrexia or infection, such as neurodegeneration, heart failure or widespread cutaneous lesions. The distribution of skin lesions presented in this case were associated with areas of relatively high surface temperature such as flexion creases and upper back. This may indicate local accumulation of toxic cofactors contributing to detachment of the epidermis from dermis. This article aims to increase awareness of this rare disease through documentation of the associated cutaneous manifestations. These present a diagnostic challenge to clinicians emphasising the need for the early identification and management of infection in patients with NAXD deficiency.

## Consent

Written informed consent was obtained from the patients’ parents for publication of this case report and accompanying images. A copy of the written consent is available for review by the Editor-in-Chief of this journal on request.

## Ethical approval

Not required.

## Funding

This Paper was funded by the 10.13039/501100000265MRC grant - MRC reference number MR/N002431/1.

### Provenance and peer review

Not commissioned, externally peer-reviewed.

## Declaration of competing interest

None.

## Registration of research studies

1. Name of the registry: N/A
2. Unique Identifying number or registration ID: N/A
3. Hyperlink to your specific registration (must be publicly accessible and will be checked): N/A
